# Screening of potential donors for anaerobic stress tolerance during germination in rice

**DOI:** 10.3389/fpls.2023.1261101

**Published:** 2023-11-10

**Authors:** Lupakisyo Mwakyusa, Maria Cristina Heredia, Newton Lwiyiso Kilasi, Richard R. Madege, Max Herzog, Shalabh Dixit

**Affiliations:** ^1^ Department of Crop Science and Horticulture, College of Agriculture, Sokoine University of Agriculture, Morogoro, Tanzania; ^2^ Department of Research and Innovation, Tanzania Agricultural Research Institute, Kigoma, Tanzania; ^3^ Rice Breeding Innovations Department, International Rice Research Institute, Los Baños, Laguna, Philippines; ^4^ Department of Biology, University of Copenhagen, Copenhagen, Denmark

**Keywords:** flooding, rice, germination, flood tolerance, genotypes

## Abstract

The rising cost of transplanting rice has made direct seeding an affordable alternative for rice establishment, particularly in Africa. However, direct seeding, while cost-effective, faces crop establishment challenges due to flooding. Uncontrolled water, driven by erratic rains in low-lying areas or uneven fields, limit germination. Rice possesses the unique ability of anaerobic germination, enabling it to sprout and emerge in oxygen-deprived conditions. Understanding rice’s response to anaerobic stress during germination is crucial for resilience breeding. Africa, although relying on direct seeding, has made limited progress in addressing flooding during germination compared to Asia. Anaerobic stress tolerance ensures successful crop emergence even in oxygen-limited environments and can help suppress weeds, a significant challenge in direct-seeded rice cultivation. This study aims to contribute by screening for potential rice donors exhibiting anaerobic stress tolerance. We screened 200 rice genotypes at Sokoine University of Agriculture (SUA) in Morogoro, Tanzania, primarily focusing on landraces with untapped potential. Using an alpha lattice design, we conducted two anaerobic experiments in September and October 2022, adding 7 cm of standing water immediately after dry seeding for flooded and maintaining a 2 cm water level after germination in the control for duration of 21 days. We identified potential donors based on selection index computed from genomic estimated breeding values (GEBVs) using eight variables: germination at 14 DAS, germination at 21 DAS, seedling height at 14 DAS, seedling height at 21 DAS, shoot dry matter at 21 DAS, root dry matter at 21 DAS, culm diameter at 21 DAS, and root length at 21DAS. Ten genotypes emerged as the most promising, exhibiting at least 70% germination in floodwater at 21 DAS and greater selection indices. These genotypes were like: Afaa Mwanza 1/159, Rojomena 271/10, Kubwa Jinga, Wahiwahi, Magongo ya Wayungu, Mpaka wa Bibi, Mwangaza, Tarabinzona, IB126-Bug 2013A, and Kanamalia with respective percentages of 75, 74, 71, 86, 75, 80, 71, 80, 70, and 73. These findings contribute to global efforts to mitigate the impacts of flooding during germination. These donors, will be potential to enrich the gene pool for anaerobic germination, providing valuable resources for breeding for flooding tolerance.

## Introduction

Flooding poses a significant threat to rice cultivation in lowland areas, impacting crop germination, growth and yield significantly (Septiningsih et al., 2009). Various forms of flooding stress, such as flooding during germination, submergence during the vegetative stage, prolonged water stagnation, deepwater conditions, and floating, are common challenges faced by rice farmers (Ismail et al., 2012). In Africa, where a considerable portion of rice cultivation relies on direct seeding, the issue of poor germination due to flooding is particularly devastating (Kuya et al., 2019). This problem is exacerbated by unpredictable weather patterns, including irregular rainfall, which affects nearly 38% of the African rice cultivation area, primarily rainfed lowlands (Diagne et al., 2013). Farmers in Africa predominantly opt for direct seeding due to its convenience in terms of crop establishment, labour efficiency, and cost-effectiveness for water management (Kuya et al., 2019). However, this practice is highly susceptible to early floods, which create an anaerobic environment that disrupts the typical aerobic ATP production necessary for germination (Hartman et al., 2020). Under anaerobic conditions, ATP production is significantly reduced, hindering normal germination and robust seedling emergence. Nevertheless, certain rice cultivars have evolved unique adaptations that exhibit relative higher starch metabolism to fuel embryo growth and coleoptile elongation under these low-oxygen conditions (Pucciariello, 2020).

Limited information is available regarding anaerobic germination within the African context, with only a handful of studies shedding light on this aspect of rice cultivation in Africa. Notably, Agbeleye et al. (2019) screened nearly 2000 *O. glaberrima* accessions using a 10 cm water depth at the Africa Rice Center research station, situated at the International Institute of Tropical Agriculture (IITA) in Ibadan, Nigeria. This study successfully identified TOG 5980, TOG 5485, TOG 5505, TOG 16704, and TOG 8347 as promising breeding resources with significant survival rates under anaerobic conditions. Another study by Asante et al. (2021) contributed to this field by identifying five genotypes, namely OBOLO, ART68-12-1-1-B-B, ART64-31-1-1-B-B, CRI-1-21-5-12, and CRI-Enapa, which exhibited at least 70% survival when subjected to screening in 10 cm water depth, out of a pool of 100 rice genotypes. While reports on anaerobic germination in Africa remain scarce, it is worth noting that this area of study has seen more comprehensive exploration on a global scale, particularly in Asia. Numerous experiments have been conducted, encompassing assessments of cultivar survival during germination, coleoptile elongation, and the identification of quantitative trait loci (QTLs).

### Top of Form

Jiang et al. (2004) identified five QTLs (qAG-1, qAG-2, qAG-5a, qAG-5b, and qAG-7) associated with anaerobic germination using recombinant inbred lines (RILs) derived from a cross between Kinmaze (japonica) and DV85 (indica). Moreover, Jiang et al. (2006) expanded on this by uncovering two QTLs (qAG-5 and qAG-11) related to anoxia tolerance using F2 populations of USSR5 and N22. Angaji (2008) furthered our knowledge by evaluating 150 BC2F2 progenies of Khaiyan and IR64. His study observed a continuous distribution of germination ranging from 0 to 68%, identifying four QTLs (qAG-1, qAG-2, qAG-11, and qAG-12) contributing to 12% to 29.24% of the phenotypic variation. Angaji et al. (2010) screened over 8000 rice accessions and revealed several cultivars with significant survival including Khaiyan, Khao Hlan On, Cody, Dholamon 64-3, Liu-Tiao-Nuo, Ma-Zhan Red, Sossoka, Kaolack, Kalongchi, and Nanhi. Notably, Khao Hlan On was further studied through crossing with IR64. Through QTL analysis on their BC2F2, five QTLs responsible for flooding tolerance, with qAG-9-2 having the most substantial effect, accounting for 33.5% of the variation. Septiningsih et al. (2013) delved into QTL mapping with F2:3 families of Ma Zhan Red and IR42, leading to the discovery of several QTLs (qAG-2, qAG-5, qAG-6, qAG-7.1, qAG-7.2, qAG-7.3, qAG-9, and qAG-12) governing AG tolerance. Among these QTLs, qAG-7.1 contributed the most, explaining 31.7% of the phenotypic variation.

Similarly, Baltazar et al. (2014) observed survival rates ranging from 3% to 90% using 300 F2:3 lines resulting from a cross between IR64 and Nanhi. A major QTL (qAG-7) was identified on chromosome 7, accounting for 22.3% of the phenotypic variation. Kim and Reinke (2018) revealed three QTLs (qAG-1a, qAG-1b, and qAG-8) linked to anaerobic germination from a population following a cross between Tai Nguyen and Anda. Baltazar et al. (2019) identified four AG-conferring QTLs (qAG3, qAG7.1, qAG7.2, and qAG7.3) through genotyping 190 F2:3 lines from IR64/Kharsu 80A, displaying a phenotypic variance ranging from 8.1% to 12.6%. Recently, Yang et al. (2022) pinpointed OsTPP1, a candidate gene located in locus 3 of chromosome 2, using RILs derived from a cross H335 and CHA-1 with significant upregulation during anoxia in tolerant lines. A significant milestone thus far has been the identification and cloning of the trehalose-6-phosphate phosphatase gene, OsTTP7, from the major QTL qAG-9-2 (Kretzschmar et al., 2015). This gene plays a vital role in sugar regulation, promoting germinating embryo growth and coleoptile elongation, ultimately leading to successful crop establishment. The emphasis on coleoptile elongation has emerged as a crucial determinant for rice survival under flooding and is essential for screening (Ismail et al., 2012; Hsu and Tung, 2015; Zhang et al., 2017; Kuya et al., 2019; Pucciariello, 2020; Su et al., 2021; Thapa et al., 2022).

Tanzania, one of the largest rice producers in Africa and second only to Madagascar in the Eastern, Central, and Southern African region (Boniphace et al., 2015), extensively relies on direct seeding for rice cultivation. The country plays a vital role as a food supplier, even catering to neighbouring nations (Andreoni et al., 2021). Regrettably, Tanzania is prone to flooding, particularly in its low-lying rice cultivation regions, which account for a substantial 71% of rice cultivation (URT, 2019). Despite its significant contribution, rice productivity in Tanzania remains low, averaging 2.3 tons per hectare (URT, 2021), and significantly lower during severe weather events. Major rice-growing valleys experience a further drop in productivity, falling to less than 2 tons per hectare in the face of unexpected erratic rainfall and floods (Kwesiga et al., 2019). Several surveys have highlighted the escalating risks of flooding in Tanzania’s rainfed lowlands, posing a threat to the country’s food security (Balama et al., 2013; Mugula and Mkuna, 2016; Brüssow et al., 2019; Ires, 2021). Remarkably, none of these studies have reported any screening aimed at assessing flooding tolerance, including anaerobic germination, within the dominant direct seeding system. Consequently, to unravel the intricacies of anaerobic germination and make a meaningful contribution to both African and global research in this field, continuous screening of diverse germplasm is deemed crucial to identify robust donors. Therefore, our study endeavours to make this contribution to this field by identifying new donors from an untapped population.

## Materials and methods

### Characteristics of the study site

The screening experiments were conducted in September and October 2022 in the screenhouse at the Sokoine University of Agriculture (SUA) crop museum in Morogoro, Tanzania. To maintain suitable conditions, fans and shade nets were installed in the screenhouse to partially control temperature and prevent water overheating. Daily air and water temperature measurements were taken at 7:00 AM and 1:00 PM (**Supplementary Figure S1A, B**). The screen house was equipped with tap water, which had a pH reading of 6.93 and an electrical conductivity (EC) of 0.08 mS/cm, as measured using a HI991301 Multimeter. The soil used was obtained from the SUA model training farm, located at coordinates 6°50’35.68272’’S and 37°39’7.65324’’E, and its characteristics are detailed in **Supplementary Table S1**.

### Germplasm

The study consisted a total of 200 test genotypes, predominantly comprising landraces (104), lines (61), and improved varieties (35). These genetic materials were primarily sourced from the International Rice Research Institute (IRRI) centers in Dakawa and Burundi, with a few obtained directly from rice farmers in Tanzania’s rice-growing regions (**Table S2**). Additionally, IRRI Headquarters contributed eight checks, categorized as tolerant, including Ciherang Sub 1 AG1, Khao Hlan On, Ma Zhan Red, Ciherang Sub1 AG1 AG2, and susceptible, such as IR64, Swarna Sub1, FR 13A, and Ciherang Sub1. To clarify the source, we included accession numbers for the formally recognized and catalogued genotypes in the International Rice Genebank Collection (IRGC), Genetic Stocks Oryza (GSOR) collection and West Africa Rice Breeding (WAB). Unfortunately, the majority of the germplasm consists of traditional landraces primarily known to local farmers. Consequently, only the formally recognized genotypes are presented with both accession numbers and accession names, while the rest are listed with accession names only.

### Phenotyping for tolerance to anaerobic stress during germination

Phenotyping for tolerance to anaerobic stress during germination was conducted following the 2021 IRRI Phenotyping protocols for abiotic stress tolerance in rice (https://books.google.co.uk/books?id=dwMlEAAAQBAJ), with some minor modifications in screening structures, flood water level, and data collection.

To ensure reliable results, two experiments were conducted in the same screenhouse, one in September and another in October, using a 14 x 16 alpha lattice design. Each experiment included flooded and control (aerobic/non-flooded) setups as per the recommended protocol. Instead of using concrete tables as specified in the protocol, we used plastic crates (60 x 40 x 30 cm) capable of retaining water. Two replications were established, each consisting of 16 plastic crates. Each genotype (entry) appeared once within each replication, except for the checks, which were randomly replicated three times.

We filled and levelled sieved soil up to 10 cm in plastic crates. Each genotype was represented by two rows of sown seeds, with each row containing 15 dry seeds. This adjustment was made to accommodate the limited space available, as the protocol recommended a minimum of 17 dry seeds per row. Seeds were sown at a depth of 0.5 cm, labeled, and covered. The distance between rows of the same genotype was 1.5 cm, and for rows of different genotypes, it was 2.5 cm. Each plastic crate accommodated 14 entries.

After seeding, water was carefully added to each crate to reach a 7 cm level from the soil surface, although the level was lower than the protocol recommendation of 10 cm. This water level was maintained for 21 days in the flooded setup. In the control setup, the soil was moistened to allow germination, and then a 2 cm water level above the ground was maintained for 21 days.

### Data collection

Initially, we recorded the initial number of seeds sown for each genotype immediately after seeding, as well as the number of surviving seedlings 14 and 21 days after seeding. This latter measurement, termed ‘number of seeds germinated’ in this study, referred to those seedlings that emerged above the water surface. Percentage germination (survival) served as the primary determinant for identifying flood-tolerant cultivars, and it was calculated using the formula: Percentage Germination = (number of seeds germinated per entry/total seeds sown per entry) * 100.

However, in an effort to enrich the identification of vigorous flood-tolerant donors, we expanded our data collection to include additional growth parameters. These parameters encompassed seedling height (cm), which was measured 14 and 21 days after seeding using a standard ruler. Furthermore, we assessed root length (cm) and culm diameter (mm), measuring them 21 days after sowing using a standard ruler and a digital Vernier caliper, respectively. To complete the assessment, we harvested sampled seedlings and subjected them to drying at 70°C for three days, yielding measurements of shoot dry matter (g) and root dry matter (g), which were then weighed.

### Sample collection and genotyping

To obtain DNA samples for genotyping, rice seeds were pre-germinated for three days and then transplanted into trays filled with a nutrient solution for 21 days of growth. Leaf samples, crucial for DNA extraction, were collected using a 6 mm diameter leaf puncher, with four leaf discs taken from each rice genotype, selected from young, healthy leaves. Subsequently, the leaf discs were freeze-dried in preparation for DNA extraction and genotyping. Genotyping was conducted using the 1k-RiCA (1k Rice Custom Amplicon Assay), a designed assay based on Illumina’s TruSeq Custom Amplicon 384 Index Kit technology (Arbelaez et al., 2019). This assay includes one thousand strategically selected Single Nucleotide Polymorphisms (SNPs) from the Cornell_6K_Array_Infinium_Rice and the 3,000 rice genomes datasets (Thomson et al., 2017; Wang et al., 2018). It is an economical method for amplification and sequencing, known for its outstanding precision and efficiency, particularly in populations of the indica subspecies. Furthermore, this assay enhances genomic predictions. The genotyping process was carried out by AgriPlex Genomics in Cleveland, Ohio, United States of America, using the plexseq method following the plexseq workflow, which includes assay design and genotype calling. All SNPs were genotyped across the samples simultaneously. Subsequently, SNP analysis was performed using a Custom SNP-calling Pipeline to identify the variants, which were then aligned to the reference Nipponbare rice genome MSU7 version (Kawahara et al., 2013).

### Analysis workflow

The data quality was assessed by subjecting them to the Bonferroni test, which helped detect and remove outliers. Phenotypic data, genotypic marker data, and QTL data were formatted to facilitate straightforward analysis. The analysis involved single experiment analysis (SEA), and multi-environment experiment (MEA) analysis using LMMsolver in R package (Boer and van Rossum, 2021).

For single experiment analysis, cleaned phenotypic data was used to produce the best linear unbiased predictions (BLUPs) for each trait in each experiment using a linear mixed model of y = β_0_ + β_1_ * r + u + b + ϵ.

Where:

y is the response variable,

β_0_ is the intercept,

β_1_ is the coefficient associated with the fixed effect of replications,

r is the fixed effect variable for replications,

u represents the random effect variable for genotype,

b represents the fixed effect variable for blocks within replications,

ϵ is the residual term that captures the unexplained variation.

The data from experiment 1 and 2 for anaerobic conditions were analysed together similar to the analysis across aerobic conditions and BLUPs were calculated in each environment. Similarly, the MEA analysis used data from the single experiments in the two environments to produce BLUPs for each trait in each genotype using a model of y = β_0_ + β_1_ * E + β_2_ * r + β_3_ * B + u + ϵ.

Where:

y is the response variable,

β_0_ is the intercept,

E is the fixed effect variable for the two environments,

β_1_ is the coefficient associated with the fixed effect of the environment,

r is the fixed effect variable for the replications,

β_2_ is the coefficient associated with the fixed effect of the replication,

B is the fixed effect variable for the blocks within replications,

β_3_ is the coefficient associated with the fixed effect of the block,

u represents the random effect variable for genotype,

ϵ is the error term.

Subsequently, cleaned genotypic marker data were analyzed to generate the genomic relationship matrix (GRM) (Endelman, 2011). Genomic estimated breeding values (GEBVs) were generated based on genetic evaluations incorporating the SEA and GRM. Additionally, phenotypic correlations were assessed using Pearson correlation coefficient and heritability was calculated as ratio of genetic variance to total variance using ASRelm. Finally, selection indices were calculated using the GEBVs and the weighted values for each trait. The index consolidated the GEBVs of all traits for a genotype into a single value, assigning weights based on their relative importance for anaerobic germination (Ceron-Rojas and Crossa, 2018). Similar weights were used for selection under aerobic and anaerobic conditions. The scales were specified as: (a) % germination 14 DAS: 10; (b) % germination 21 DAS: 8; (c) seedling height 14 DAS: 5; (d) seedling height 21 DAS: 4; (e) shoot dry matter 21 DAS: 7; (f) root dry matter 21 DAS: 4; (g) culm diameter 21 DAS: 3; and (h) root length: 5.

Therefore, the selection of high-surviving genotypes was based on the selection index across all measured traits and germination (BLUPs) 21 days after sowing (DAS). The selection criteria applied were summarized as follows: Selection of tolerant genotypes to anaerobic conditions: Genotypes with the highest selection index ≥ 25 and germination 21 DAS ≥ 70%. Selection of the best-performing genotypes under aerobic conditions: Genotypes with the highest selection index ≥ 25 and germination 21 DAS ≥ 80%. Selection of the best-performing genotypes across experiments (anaerobic-aerobic): Genotypes with the highest selection index ≥ 25 and germination 21 DAS ≥ 80%.

### The use of genomic estimated breeding values and selection index

The study employed Genomic Estimated Breeding Values (GEBVs) to identify robust donor lines. Unlike the more common Genome-Wide Association Study (GWAS), which typically requires a larger number of Single Nucleotide Polymorphisms (SNPs) and focuses on a few significant GWAS peaks, our approach utilized a limited number of markers. This allowed us to comprehensively assess the overall value of each line as potential parents. To enhance the selection process, we calculated a selection index for each genotype, incorporating eight traits with predefined weights (relative economic weights) assigned to each trait. This was done using an equation adapted from Pešek and Baker (1969), given as: $\text{I}=\sum_{i=1}^{n}b_{i}x_{i}$ where, “I” is the selection index, b_i_ is the weight for the i-th trait, and x_i_ is the phenotypic value of the i-th trait. These weights were primarily based on each trait’s contribution to anaerobic stress tolerance. The most critical traits, percentage germination (survival) at 14 and 21 days after seeding (DAS), received the highest weights. These traits are commonly used as key indicators for selecting lines adapted to anaerobic stress (Septiningsih et al., 2013; Baltazar et al., 2019; IRRI, 2021). Following these, shoot dry matter received a relatively high weight, followed by seedling height at 14 DAS, which had a weight similar to that of root length. Seedling height at 21 DAS and root dry matter were assigned equal weights, while culm diameter received the lowest weight.

## Results

### Genotypic scores and genomic predictions

The 208 rice genotypes subjected to genotyping passed the quality control threshold, surpassing a 66% pass rate and achieving a successful call rate of 93.9%. The sample mean heterozygosity was 2.3%, while poor data for samples and markers was 1.4% and 7.9% respectively. However, the identification of variants harbouring AG1, a QTL known for conferring tolerance to anaerobic stress during germination and included in 1K RiCA, was limited due to its low coverage. The call rate for the AG1 QTL, indicating QTL absence, was 36.1%, while the uncall rate reached 64.9%. The study observed that none of the test genotypes scored as positive for AG1 QTL availability. Notably, the high-performing genotypes that exhibited tolerance to flooding stress in this study were predominantly uncalled for AG1 QTL. Moreover, the study observed a wide range of Genomic Estimated Breeding Values (GEBVs) for the traits assessed among the cultivars under flooded conditions. This diversity greatly facilitated the selection process (**Table S3**). For germination 14 DAS, GEBVs ranged from -4.98 to 74.58%, while germination 21 DAS showed values between 9.77 and 87.91%. Seedling height at 14 DAS displayed variations from 10.78 to 21.98 cm, and at 21 DAS, it ranged from 19.46 to 40.98 cm. Culm diameter exhibited GEBVs from 1.65 to 1.68 mm, root length from 5.09 to 7.35 cm, shoot dry matter from 0.01 to 0.04 g, and root dry matter from 0.003 to 0.01 g.

### Phenotypic performance

**Figure 1:**
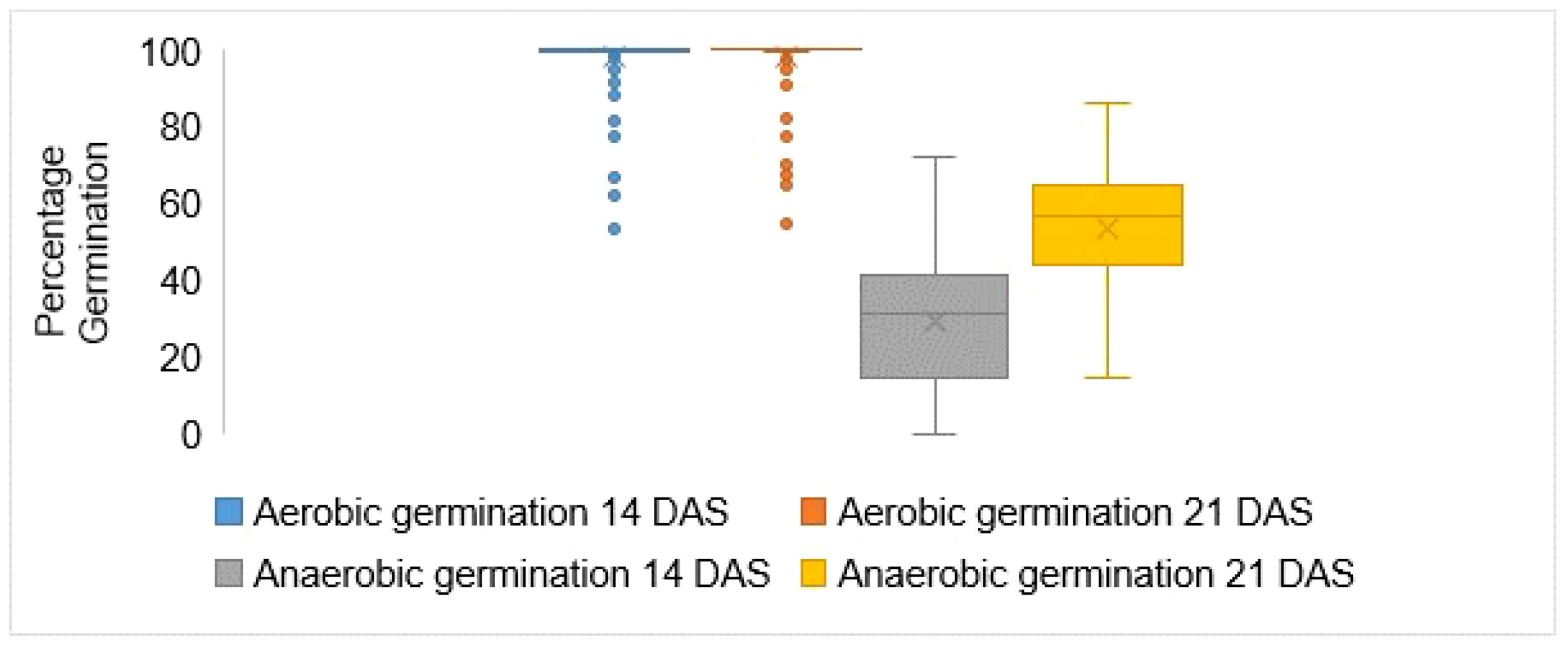
Germination response of rice cultivars to anaerobic stress compared to aerobic condition using BLUPs. DAS indicates days after seeding, and BLUPs stands for best linear unbiased predictions.

There was a wide variation in germination rates among the screened rice genotypes under anaerobic conditions, ranging from 0% to 100%. In contrast, most rice genotypes under control conditions exhibited nearly 100% germination. In Experiment 1, the germination rate at 14 days after seeding (DAS) was 98.2% under control conditions but dropped significantly to 29.2% under anaerobic conditions. Notably, several genotypes, including Mpaka wa Bibi, Kanamalia, Tarabinzona, and Wahiwahi, exhibited strong early emergence with germination rates of 74.1%, 76.9%, 82.1%, and 86%, respectively. Among the checks, the known tolerant check, Ciherang Sub1 AG1 AG2, displayed higher germination rates compared to other checks (**Table S4**). In Experiment 2, the aerobic germination rate was 98.4%, while the anaerobic germination rate was slightly lower at 31.1%. Germination rates increased at 21 DAS under anaerobic conditions compared to 14 DAS. In Experiment 1, seedling emergence increased from 29.2% at 14 DAS to 59.8% at 21 DAS, indicating a 30.6% increase. In Experiment 2, seedling emergence increased from 31.1% at 14 DAS to 47.5% at 21 DAS, indicating a 16.4% increase. Control (aerobic) conditions maintained consistent germination rates of 98.2% and 98.4% at 14 and 21 days after seeding, respectively. Overall, the anaerobic experiments displayed an emergence rate of 29.4% at 14 DAS, significantly lower than the control with 98.3%. Some specific genotypes, including BG90-2, IR117842-11-1RGA-1RGA-1RGA-2, IR15T1302, TXD 307, and Yunyin, failed to emerge from the water at 14 days after seeding. At 21 DAS, seedling emergence in the anaerobic experiments was 53.7%, compared to 98.4% in the non-flooded condition. Germination rates ranged from 14.4% to 85.9% in the flooded condition, while in the control condition, they ranged from 54.5% to 100% (**Figure 1**). Additionally, some outstanding germination was displayed by Wahiwahi, Tarabinzona, and Mpaka wa Bibi. Wahiwahi attained the highest germination rate of 86% under flooding stress, followed by Tarabinzona and Mpaka wa Bibi, each with 80% germination at 21 DAS. These genotypes outperformed the tolerant check Ciherang Sub1 AG1 AG2, which had a germination rate of 79%.

Other traits, including seedling height, root length, shoot dry matter, root dry matter, and culm diameter, were also significantly affected by the anaerobic condition. In Anaerobic Experiment 1, the average seedling height at 14 DAS was 13.4 cm lower than that in the control, which had a seedling height of 25.9 cm. In Experiment 2, seedling height was 17.6 cm lower in the flooded environment compared to the control, which had a height of 31.6 cm. At 21 days after seeding, seedling heights in the anaerobic experiments remained lower compared to their respective control setups. The average seedling height in the anaerobic experiments was 15.6 cm and 32.4 cm at 14 and 21 DAS, respectively, while their controls had heights of 28.8 cm and 44.1 cm. Root growth in the anaerobic experiments was restricted, with a length of 6.5 cm under anaerobic conditions compared to 9.0 cm in non-flooded conditions. Seedling dry matter, including shoot and root dry matter, was lower in the anaerobic experiments compared to the dry matter in their control setups. In the anaerobic experiments, the average shoot dry matter was 0.025 g, while it was 0.071 g in the control. The average root dry matter was 0.071 g in the anaerobic experiments, whereas the control setups had 0.014 g. Additionally, culm diameter was also affected by floods, displaying an average diameter of 1.7 mm compared to 2.7 mm in the aerated environments. Detailed information regarding phenotypic performance is provided in **Table 1**.

**Table 1:** Overall summary for Phenotypic Performance of Rice Genotypes for Various Traits under Anaerobic Conditions Compared to Control (Aerobic) Conditions.

Experiment	Traits	Minimum	Mean	Maximum	Reliability
Anaerobic Experiment 1	GERMINATION (14DAS)-(%)	0.0	29.2	86.0	0.78
	GERMINATION (21DAS)-(%)	0.4	59.8	100.0	0.64
	Seedling Height (14DAS)-(cm)	7.5	13.4	27.0	0.66
	Seedling Height (21DAS)-(cm)	9.6	28.3	38.0	0.76
	Culm Diameter -(mm)	1.1	1.4	1.6	0.34
	Root Length-(cm)	2.4	5.3	9.6	0.29
	Shoot Dry matter-(g)	0.003	0.019	0.034	0.66
	Root Dry matter-(g)	0.001	0.005	0.012	0.52
Anaerobic Experiment 2	GERMINATION (14DAS)-(%)	0.0	31.1	87.7	0.80
	GERMINATION (21DAS)-(%)	0.0	47.5	93.7	0.77
	Seedling Height (14DAS)-(cm)	8.5	17.6	32.8	0.51
	Seedling Height (21DAS)-(cm)	18.4	36.5	49.2	0.79
	Culm Diameter -(mm)	1.1	1.9	2.5	0.55
	Root Length-(cm)	1.2	7.7	13.1	0.55
	Shoot Dry matter-(g)	0.004	0.030	0.057	0.59
	Root Dry matter-(g)	0.000	0.007	0.018	0.44
Aerobic (control) Experiment 1	GERMINATION (14DAS)-(%)	38.3	98.2	100.0	0.93
	GERMINATION (21DAS)-(%)	38.3	98.2	100.0	0.93
	Seedling Height (14DAS)-(cm)	14.3	25.9	36.0	0.91
	Seedling Height (21DAS)-(cm)	23.1	38.5	52.0	0.90
	Culm Diameter -(mm)	1.6	2.0	2.2	0.64
	Root Length-(cm)	4.5	8.7	14.1	0.34
	Shoot Dry matter-(g)	0.004	0.030	0.057	0.85
	Root Dry matter-(g)	0.003	0.012	0.022	0.48
Aerobic (control)Experiment 2	GERMINATION (14DAS)-(%)	54.4	98.1	100.0	0.87
	GERMINATION (21DAS)-(%)	60.8	98.4	100.0	0.89
	Seedling Height (14DAS)-(cm)	19.8	31.6	42.3	0.86
	Seedling Height (21DAS)-(cm)	31.1	49.7	63.1	0.86
	Culm Diameter -(mm)	2.0	3.4	4.7	0.66
	Root Length-(cm)	4.3	9.3	14.9	0.39
	Shoot Dry matter-(g)	0.026	0.092	0.153	0.78
	Root Dry matter-(g)	0.007	0.016	0.029	0.59
Across Anaerobic Experiments	GERMINATION (14DAS)-(%)	0.0	29.4	72.4	
	GERMINATION (21DAS)-(%)	14.4	53.7	85.9	
	Seedling Height (14DAS)-(cm)	12.6	15.6	21.8	
	Seedling Height (21DAS)-(cm)	19.9	32.4	40.2	
	Culm Diameter -(mm)	1.6	1.7	1.7	
	Root Length-(cm)	5.7	6.5	7.6	
	Shoot Dry matter-(g)	0.015	0.025	0.036	
	Root Dry matter-(g)	0.003	0.007	0.013	
Across Aerobic (control) Experiments	GERMINATION (14DAS)-(%)	53.1	98.3	100.0	
	GERMINATION (21DAS)-(%)	54.5	98.4	100.0	
	Seedling Height (14DAS)-(cm)	18.3	28.8	37.0	
	Seedling Height (21DAS)-(cm)	30.3	44.1	54.8	
	Culm Diameter -(mm)	2.5	2.7	2.8	
	Root Length-(cm)	8.3	9.0	9.6	
	Shoot Dry matter-(g)	0.042	0.071	0.102	
	Root Dry matter-(g)	0.008	0.014	0.020	
Across (aerobic-anaerobic)Experiments	GERMINATION (14DAS)-(%)	30.8	63.9	79.7	
	GERMINATION (21DAS)-(%)	39.4	76.1	85.9	
	Seedling Height (14DAS)-(cm)	12.5	21.9	30.1	
	Seedling Height (21DAS)-(cm)	25.7	38.2	47.6	
	Culm Diameter -(mm)	2.0	2.2	2.3	
	Root Length-(cm)	6.6	7.8	9.0	
	Shoot Dry matter-(g)	0.028	0.048	0.068	
	Root Dry matter-(g)	0.006	0.010	0.016	

### Association of traits

**Figure 2:**
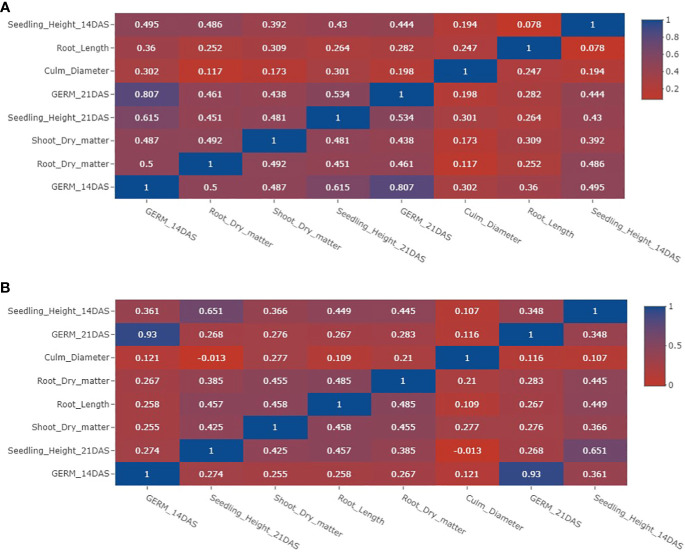
Phenotypic correlations of traits under anaerobic and control experiments. **(A)** Phenotypic correlations of traits for anaerobic experiments. **(B)** Phenotypic correlations of traits for aerobic (control) experiments. DAS indicates days after seeding.

A very strong positive correlation was evident between germination at 14 days after seeding (DAS) and germination at 21 DAS, irrespective of whether the conditions were flooded or under control (**Figure 2**). Additionally, significant positive correlations observed between traits such as germination at 14 DAS and seedling height at 21 DAS (r = 0.615) in flooded conditions, as well as between seedling height at 14 DAS and 21 DAS (r = 0.615) in control conditions. In contrast, the correlation between culm diameter and other traits appeared generally weak, indicating a limited relationship between culm diameter and the other recorded traits.

### Broad sense heritability

The study revealed high heritability for germination, seedling height, and shoot dry matter, even in the presence of flooding stress. The trait with the highest heritability (H^2 ^= 0.71) was germination 14 days after seeding, which was consistently maintained in both experiments 1 and 2 (**Table 2**). Seedling height at 21 days after seeding, germination at 21 days after seeding, and shoot dry matter also displayed relatively high heritability. However, certain traits such as root length, culm diameter, and root dry matter exhibited low heritability. Notably, among all the recorded traits, root length displayed the lowest heritability.

**Table 2:** Broad Sense Heritability for studied traits in anaerobic stress conditions.

Traits	Anaerobic Experiment 1	Anaerobic Experiment 2
Germination (14DAS)-(%)	0.71	0.71
Germination (21DAS)-(%)	0.61	0.69
Seedling height (14DAS)-(cm)	0.57	0.42
Seedling height (21DAS)-(cm)	0.67	0.7
Culm diameter -(mm)	0.31	0.48
Root length-(cm)	0.26	0.46
Shoot dry matter-(g)	0.59	0.53
Root dry matter-(g)	0.48	0.44

DAS indicates days after seeding.

### Effects of anaerobic stress on germination and growth of direct-seeded rice

**Figure 3:**
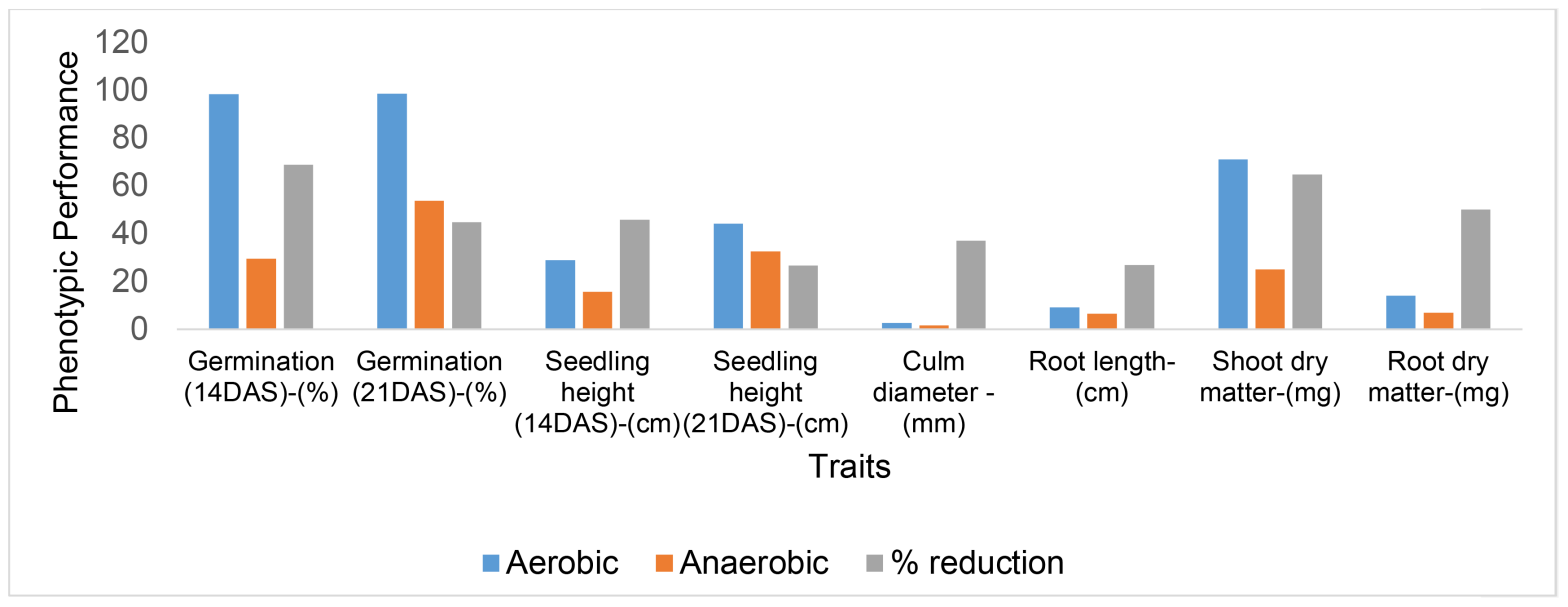
Quantification of the effects of flooding on germination and growth of direct-seeded rice. DAS indicates days after seeding. Dry matter values were converted from grams (g) to milligrams (mg) for improved visualization of the graph.

The effects of anaerobic stress on the germination and growth of direct-seeded rice were assessed by comparing the anaerobic (flooded) condition with a control condition (non-flooded or aerobic). The effects of the stress on various traits were quantified as percentage reduction (**Figure 3**). Overall, the anaerobic stress had severe effects on certain traits, particularly germination 14 days after seeding (DAS), shoot dry matter, and root dry matter compared to the control condition. In contrast, lesser effects were observed for traits such as seedling height 21 DAS and root length. Notably, parameters like germination and seedling height were significantly affected at 14 DAS than 21 DAS.

### Selection of donors adapted to anaerobic stress during germination

Ten test entries have emerged as promising candidates for anaerobic stress adaptation during germination, primarily due to their robust selection index. These entries not only exhibited substantial best linear unbiased predictions (BLUPs) for germination percentage at 21 days after seeding (DAS) but also displayed vigorous emergence in flooded conditions, maintaining a survival rate of at least 70 (**Table 3**). Interestingly, our study revealed exceptional performance in both aerobic and aerobic-anaerobic conditions, with thirteen genotypes excelling in the former and seventeen entries displaying remarkable performance across environments, despite the primary focus of our study being anaerobic stress tolerance. These genotypes displayed the largest selection index values and consistently achieved germination percentages of no less than 80% at 21 DAS. For a more comprehensive overview of the genomic estimated breeding values (GEBVs) and selection indices of all 208 genotypes, refer to **Table S4**. In contrast, among the checks employed in our study, Ciherang Sub1 AG1 AG2 stood out with the highest germination percentage of 79% when seeded under flooded conditions at 21 DAS. It’s worth noting that the germination rates of the other checks fell below the 70% threshold. Consequently, Ciherang Sub1 AG1 AG2 was the sole known check among our selections that exhibited robust germination performance.

**Table 3:** List of potential donors best suited under anaerobic stress during germination.

Genotype	Condition	Selection Index	Germination percentage 21 DAS	Remarks
Afaa Mwanza 1/159	Anaerobic	46	75	Entry
Ciherang Sub1 AG1 AG2	Anaerobic	43	79	Check
Rojomena 271/10	Anaerobic	31	74	Entry
Kubwa jinga	Anaerobic	30	71	Entry
Wahiwahi	Anaerobic	28	86	Entry
Magongo ya Wayungu	Anaerobic	28	75	Entry
Mpaka wa bibi	Anaerobic	26	80	Entry
Mwangaza	Anaerobic	26	71	Entry
Tarabinzona	Anaerobic	25	80	Entry
IB126-Bug 2013a	Anaerobic	25	70	Entry
Kanamalia	Anaerobic	25	73	Entry
Iron	Aerobic	116.59	92	Entry
Mwangaza	Aerobic	78.65	95	Entry
IR15T1302	Aerobic	50.82	97	Entry
Tunduru	Aerobic	50.70	100	Entry
Ciherang Sub1 AG1	Aerobic	45.51	100	Check
Line-8A-2	Aerobic	37.61	100	Entry
NERICA 2	Aerobic	36.96	100	Entry
Afaa melela	Aerobic	33.61	100	Entry
IR16T1339	Aerobic	32.97	95	Entry
Lunyuki	Aerobic	32.32	100	Entry
Mbega	Aerobic	30.30	100	Entry
NERICA 4	Aerobic	29.00	100	Entry
Line-18-Niwur1	Aerobic	28.83	100	Entry
Kaling’anaula	Aerobic	28.12	100	Entry
Swarna Sub1	Aerobic	27.54	100	Check
Afaa Mwanza 1/159	Across	56	84	Entry
Ciherang Sub1 AG1 AG2	Across	54	85	Check
Kanamalia	Across	41	82	Entry
Rojomena 271/10	Across	39	83	Entry
Kaling’anaula	Across	39	80	Entry
Chimdima	Across	36	81	Entry
Pishori-Brown	Across	34	81	Entry
Gigante	Across	33	82	Entry
Tarabinzona	Across	28	84	Entry
Faya dume 3	Across	28	82	Entry
Faya dume 1	Across	26	83	Entry
Tosa	Across	26	81	Entry
Magongo ya Wayungu	Across	25	83	Entry
Ringa	Across	25	81	Entry
Mpaka wa bibi	Across	25	84	Entry
IB126-Bug 2013a	Across	25	81	Entry
Kivuli	Across	25	80	Entry
Mzungu	Across	25	81	Entry

Exceptional results for aerobic condition and across aerobic-anaerobic environments are also included. The selection was based on a selection index generated from GEBVs (Genomic Estimated Breeding Values) for multiple traits and BLUPs (Best Linear Unbiased Predictions) for germination 21 DAS (days after seeding), as described in the methodology.

## Discussion

### Variation in performance

Anaerobic germination is a unique characteristic of rice seeds that allows the initiation of the germination process and subsequent emergence despite unfavourable flooding stress (Ghosal et al., 2019; Panda and Barik, 2021). This study observed diverse responses of genotypes to anaerobic stress during germination and early seedling growth. Genotype-specific responses to oxygen deficiency during the germination stage have also been reported by Ray et al. (2016), suggesting the importance of varying responses for the selection of tolerant lines. Tolerant genotypes that survive under limited oxygen conditions are considered potential candidates for anaerobic germination (Ray et al., 2016). Maintaining a relatively high metabolism has been identified as advantageous for surviving anaerobiosis (Ismail et al., 2009). Therefore, tolerant cultivars had increased expression of α-amylase triggered by low sugar levels, unlike intolerant cultivars (Ismail et al., 2012). Rice varieties tolerant to anaerobic stress generally exhibit higher sugar levels, while susceptible cultivars tend to have higher starch concentrations and lower sugar (Mondal et al., 2020).

In this study, the 10 potential genotypes identified exhibited significantly higher survival rates under limited oxygen conditions, characterized by increased germination, seedling growth, and biomass accumulation compared to other genotypes. Partheeban et al. (2017) also reported greater seedling vigor in highly tolerant cultivars compared to moderate and susceptible ones under anaerobic stress. Furthermore, Mondal et al. (2020) found that genotypes tolerant to anaerobic stress displayed higher amylase activities, resulting in increased germination and subsequent seedling growth. Therefore, these genotypes hold significant potential as donors in breeding for anaerobic stress tolerance.

The strong positive correlation between germination at 14 and 21 days after sowing (DAS) underscores the critical role of early emergence at 14 DAS in mitigating flooding stress and promoting successful establishment. Early germination tightly correlates with rapid seedling growth, exemplified by the strong association between seedling height at 21 DAS and emergence at 14 DAS. These findings underscore the significance of early germination and growth in mitigating the impacts of flooding. Ismail et al., 2009 observed robust establishment of tolerant rice under limited oxygen conditions due to vigorous early growth. In contrast, this study observed limited relationships between culm diameter and other studied traits, irrespective of the growing conditions. These findings imply that culm diameter is not significantly linked to changes in other traits during the early stages of germination. Furthermore, weak correlations were observed between root traits and other characteristics, particularly under anaerobic conditions. This aligns with the fact that root growth is severely constrained during flooding, as the plant directs its energy towards seedling emergence to access oxygen more efficiently. This prioritization of starch breakdown for coleoptile elongation, facilitating oxygen uptake, comes at the expense of root development. (Singh et al., 2017; Pucciariello, 2020; Hirano et al., 2023).

To enhance breeding efforts, heritability plays a crucial role that cannot be overlooked (Roy and Shil, 2020). Heritability quantifies the extent to which a trait can be inherited by offspring (Ajmera et al., 2017). The success and efficiency of breeding programs take into account not only genetic variations and genetic advancement but also heritability (Abebe et al., 2017; Adhikari et al., 2018). Hence, in this study, having prior knowledge of trait heritability is imperative. Several key traits essential for anaerobic germination, such as germination rate, seedling height, and shoot dry matter, exhibited high heritability, suggesting their potential for successful improvement. As pointed out by Demeke et al. (2022), traits with higher heritability play a significant role in determining the potential for population enhancement. Conversely, traits like root length, culm diameter, and root dry matter displayed lower to medium heritability, indicating a lesser genetic influence on these rice characteristics during flooding stress.

### Germination stage oxygen deficiency and associated impacts

Under flooding stress, oxygen diffusion is severely hindered, making it difficult for normal aerobic respiration to occur (Ma et al., 2020). The lack of oxygen becomes lethal for rice survival. However, certain rice cultivars can successfully establish themselves under low or no oxygen conditions (Ray et al., 2016). There are variations among genotypes in terms of flooding tolerance, which suggests their potential for rice establishment during anaerobic stress. In this study, variations among genotypes in germination under submergence stress were observed, with some exhibiting very low survival rates while others showed high emergence. Different cultivars respond differently to oxygen deficits, with some unable to survive and others displaying varying degrees of tolerance (Angaji et al., 2010; Thapa et al., 2022). Moreover, all the traits assessed in this study were largely affected by floods compared to the control condition. Submerged seeds experienced delayed seedling emergence, which could be attributed to interference with metabolism under anaerobic stress, resulting in low energy production. Magneschi and Perata (2009) highlighted that only 2 ATP molecules are produced from 1 molecule of glucose under anaerobic conditions, while nearly 38 molecules of ATP are produced under normal conditions. With such limited energy supply to germinating seeds, even emergence is compromised. Similarly, under restricted oxygen conditions, the normal process of root respiration is altered, affecting nutrient uptake and seedling growth (Miro and Ismail, 2013). Hartman et al. (2020) also emphasized that low energy generation due to oxygen deficit during flooding stress leads to reduced crop survival and growth.

### Candidate genotypes for flooding tolerance

Tolerant cultivars are in high demand due to their ability to maintain high germination rates and successfully overcome stress (Chamara et al., 2018). It’s impressive that the observed variation in performance among the screened rice genotypes resulted in the identification of some outstanding cultivars. Among them, Wahiwahi, Tarabinzona, and Mpaka wa Bibi displayed vigorous seedling emergence. The known anaerobic stress-tolerant check, Ciherang Sub1 AG1 AG2 (Mondal et al., 2020), exhibited slightly lower germination rates than the three genotypes. Moreover, out of the ten genotypes identified as tolerant, five were landraces. These included Kubwa Jinga, Wahiwahi, Magongo ya Wayungu, Mpaka wa Bibi, and Kanamalia. Miro and Ismail (2013) found that landraces are better adapted to flooding stress compared to modern cultivars. Mohanapriya et al. (2022) observed robust crop establishment and vigor of traditional landraces under oxygen-limited conditions. Additionally, Barik et al. (2019) noted the great potential of utilizing landraces to adapt to anaerobic stress in direct-seeded rice. Therefore, the results of this study provide a breakthrough of great importance in advancing our understanding of anaerobic germination.

## Conclusion

Successful crop establishment of rice under low or no oxygen conditions is crucial, as it enhances resilience in flood-prone areas. In this study, we observed significant variation in germination and growth attributes, underscoring their importance for selection. Notably, we identified Afaa Mwanza 1/159, Rojomena 271/10, Kubwa Jinga, Wahiwahi, Magongo ya Wayungu, Mpaka wa Bibi, Mwangaza, Tarabinzona, IB126-Bug 2013A, and Kanamalia as candidates tolerant to anaerobic stress. Some genotypes, like Tarabinzona, Mpaka wa Bibi, and Wahiwahi, exhibited the highest survival rates and germination slightly greater than the best tolerant check, Ciherang Sub1 AG1 AG2. These resources for breeding were characterized by vigorous phenotypic emergence, selected in complement with genomic value. As a result, researchers can use these valuable resources for QTL mapping with biparental or multi-parent populations. The results contribute to the limited African knowledge on flooding tolerance and are likely to strengthen efforts in resilience breeding. Therefore, the identified genotypes’ tolerance to anaerobic stress holds global significance, given the current challenging weather dynamics.

## Data availability statement

The raw data supporting the conclusions of this article will be made available by the authors, without undue reservation.

## Author contributions

LM: Conceptualization, Data curation, Formal Analysis, Methodology, Validation, Visualization, Writing – original draft. MH: Data curation, Formal Analysis, Methodology, Writing – review & editing. NK: Supervision, Writing – review & editing. RM: Supervision, Writing – review & editing. MH: Supervision, Writing – review & editing. SD: Formal Analysis, Methodology, Software, Supervision, Writing – review & editing.

## Supplementary material

Supplementary Table 2 List of genotypes characterized for anaerobic germination.

Supplementary Table 3 Genomic Estimated Breeding Values (GEBVs) and Selection Indices of 208 Rice Genotypes for Anaerobic Stress Tolerance Donor Identification. Additional information on selection for aerobic and aerobic-anaerobic conditions is provided. Note: DAS - Days After Seeding and BLUPs is for Best Linear Unbiased Predictions.

Supplementary Table 4 Phenotypic Performance of 208 Rice Genotypes for Various Traits Tested for Anaerobic Stress Tolerance During Germination compared to control (aerobic) condition.
