# Supplementary material for: Screening of potential donors for anaerobic stress tolerance during germination in rice
Source: Front Plant Sci. 2023 Nov 10;14:1261101. doi: 10.3389/fpls.2023.1261101 (PMC10667690; doi:10.3389/fpls.2023.1261101)
Supplement: Supplementary file 1 [file Table_1.docx]

**Figure S1a:** Daily Air and Water Temperature readings in the Screenhouse during Experiment 1 recorded using TZ-TempU03 and mercury thermometer at 0700 and 1300 hours respectively.

**Figure S1b:** Daily Air and Water Temperature readings in the Screenhouse during Experiment 2 recorded using TZ-TempU03 and mercury thermometer at 0700 and 1300 hours respectively.
