# Supplementary material for: Screening of potential donors for anaerobic stress tolerance during germination in rice
Source: Front Plant Sci. 2023 Nov 10;14:1261101. doi: 10.3389/fpls.2023.1261101 (PMC10667690; doi:10.3389/fpls.2023.1261101)
Supplement: Supplementary Table 2 — List of genotypes characterized for anaerobic germination. [file Table_2.docx]

**Table S1:** Soil Analysis Results for Topsoil Composite Sample from the Model Training Farm at Sokoine University of Agriculture, Representing the Soil Used in the Experiments.

| **Parameter** | **Quantity** | **Parameter** | **Quantity** |
| --- | --- | --- | --- |
| Texture | 56.32% clay | K | 2.19 Cmol^+^/Kg |
|  | 6.56% silt | Mg | 1.26 Cmol^+^/Kg |
|  | 37.12% sand | Ca | 0.29 Cmol^+^/Kg |
| pH | 5.73 | S | 12.7 mg/Kg |
| EC | 0.19 mS/cm | Cu | 2.31 mg/Kg |
| C.E.C | 3.82 Cmol^+^/Kg | Zn | 6.94 mg/Kg |
| OC | 1.15% | Mn | 81.27 mg/Kg |
| TN | 0.18% | Fe | 21.50 mg/Kg |
| P | 4.50 mg/Kg | Na | 0.06 Cmol^+^/Kg |
